# Synthesis and Biological Evaluation of Resveratrol Derivatives as Melanogenesis Inhibitors

**DOI:** 10.3390/molecules200916933

**Published:** 2015-09-17

**Authors:** Qing Liu, CheongTaek Kim, Yang Hee Jo, Seon Beom Kim, Bang Yeon Hwang, Mi Kyeong Lee

**Affiliations:** 1 College of Pharmacy, Chungbuk National University, Cheongju, Chungbuk 28644, Korea; E-Mails: liuqing7115@hotmail.com (Q.L.); qow0125@naver.com (Y.H.J.); suntiger85@hanmail.net (S.B.K.); byhwang@chungbuk.ac.kr (B.Y.H.); 2 RNS Inc., Yuseong-Gu, Daejeon 34182, Korea; E-Mail: happilion@rns.co.kr

**Keywords:** melanin, resveratrol derivatives, tyrosinase, tyrosinase-related protein (TRP)-1

## Abstract

Resveratrol (**1**), a naturally occurring stilbene compound, has been suggested as a potential whitening agent with strong inhibitory activity on melanin synthesis. However, the use of resveratrol in cosmetics has been limited due to its chemical instability and poor bioavailability. Therefore, resveratrol derivatives were prepared to improve bioavailability and anti-melanogenesis activity. Nine resveratrol derivatives including five alkyl ether derivatives with C_2_H_5_, C_4_H_9_, C_5_H_11_, C_6_H_13_, and C_8_H_17_ (**2a**–**2e**) and four ester derivatives with CH_3_, CH=C(CH_3_)_2_, CH(C_2_H_5_)C_4_H_9_, C_7_H_15_ (**3a**–**3d**) were newly synthesized and their effect on melanin synthesis were assessed. All the synthetic derivatives efficiently reduced the melanin content in α-MSH stimulated B16F10 melanoma cells. Further investigation showed that the inhibitory effect of **2a** on melanin synthesis was achieved not by the inhibition of tyrosinase activity but by the inhibition of melanogenic enzyme expressions such as tyrosinase and tyrosinase-related protein (TRP)-1. Our synthetic resveratrol derivatives have more lipophilic properties than resveratrol by the addition of alkyl or acyl chains to free hydroxyl moiety of resveratrol; thus, they are expected to show better bioavailability in skin application. Therefore, we suggest that our synthetic resveratrol derivatives might be promising candidates for better practical application to skin-whitening cosmetics.

## 1. Introduction

Melanin is produced in melanocytes by melanogenesis and determines skin and hair color. It also plays an important role in skin protection from UV radiation. However, excessive accumulation by pathological and environmental factors induces pigmentation problems and has become a critical issue in the cosmetic field [1,2]. Therefore, melanogenesis inhibitors have become important constituents in cosmetic products for depigmentation [3,4,5]. 

Melanogenesis is a complex biosynthetic process regulated by enzymatic and chemical reactions. Exposure of the skin to UV radiation stimulates keratinocytes to secrete α-melanocyte stimulating hormone (α-MSH). α-MSH then binds to melanocortin 1 receptor (MC1R) on melanocyte and induces melanogenesis. Microphthalmia-associated transcriptional factor (MITF) is a key transcriptional factor in melanogenesis that involved in the regulation of melanogenic enzymes such as tyrosinase, tyrosinase-related protein (TRP)-1 and TRP-2. Tyrosinase catalyzes the conversion of l-tyrosine to dopaquinone, the first rate-limiting step in the melanogenesis. TRP-1 and TRP-2 are also involved in the major steps of melanin synthesis. Therefore, tyrosinase, TRP-1 and TRP-2 have become important targets in the development of depigmenting agents, such as skin-whitening cosmetics [6,7,8,9].

Resveratrol, 3,4′,5-trihydroxy-*trans*-stilbene, has diverse biological activities, such as antioxidant, anti-inflammatory and cardiovascular protective effects [10,11,12]. Resveratrol also has been reported to reduce melanin synthesis [13,14,15]. However, its utilization and development in products are limited due to the chemical stability, poor solubility, and low bioavailability [16,17]. Therefore, attempts to increase the bioavailability of resveratrol have been made in many fields. Drug delivery systems using new formulations, such as encapsulation are suggested to enhance bioavailability [18,19,20]. The resveratrol derivatives, such as resveratrol dimers or synthetic derivatives, also have been reported to have better biological activity with fewer drawbacks [21,22,23,24,25]. Recently, the acetylated derivative of resveratrol has been synthesized with more efficient anti-melanogenic activity and better stability [26]. For the purpose of developing anti-melanogenesis inhibitory resveratrol derivatives with better bioavailability, nine resveratrol derivatives, five alkyl ether derivatives and four alkyl ester derivatives by chemical reaction and were synthesized in the present study. Effect on melanogenesis and the mode of action were also investigated. 

## 2. Results and Discussion

### 2.1. Chemistry

**Scheme 1:**
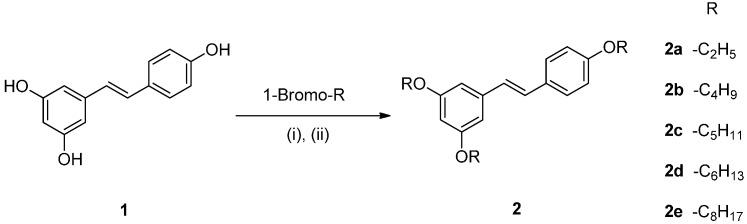
Synthesis of resveratrol ether derivatives **2a**–**e**.

**Scheme 2:**
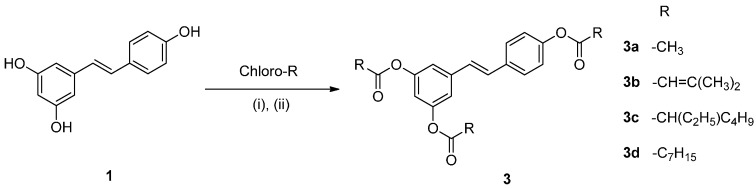
Synthesis of resveratrol ester derivatives **3a**–**d**.

**Figure 1:**
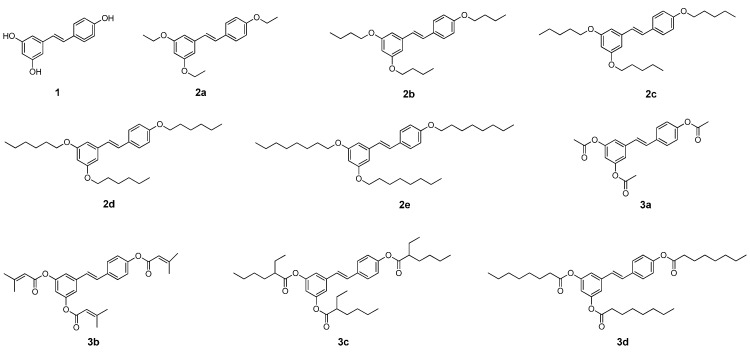
Chemical structure of resveratrol (**1**) and its derivatives (**2a**–**e** and **3a**–**d**).

The resveratrol ether derivatives were synthesized as shown in Scheme 1. Resveratrol (**1**) was converted to the corresponding ether derivatives (**2a**–**2e**) in the presence of corresponding alkyl bromides. The synthesis of resveratrol ester derivatives is shown in Scheme 2. Resveratrol ester derivatives (**3a**–**3d**) were synthesized by the addition of corresponding acyl chlorides. The structures of synthetic derivatives were confirmed by spectroscopic analysis including NMR, IR and MS analysis (Figure 1).

*Reagents and conditions*: (i) DMF, NaOH, 10 min; (ii) 1-bromoethane, 1-bromobutane, 1-bromopentane, 1-bromohexane, or 1-bromooctane, 40 °C, 24 h.

*Reagents and conditions:* (i) CH_2_Cl_2_, 10 °C, 5 min; (ii) TEA, DMAP, 10 °C; (iii) acetyl chloride, 3,3-dimethylacryloyl chloride, 2-ethylhexanoyl chloride, or octanoyl chloride, 10 °C; 1 h.

### 2.2. Effect on Melanogenesis

#### 2.2.1. Effect on Melanin Content in B16F10 Melanoma Cells

**Figure 2:**
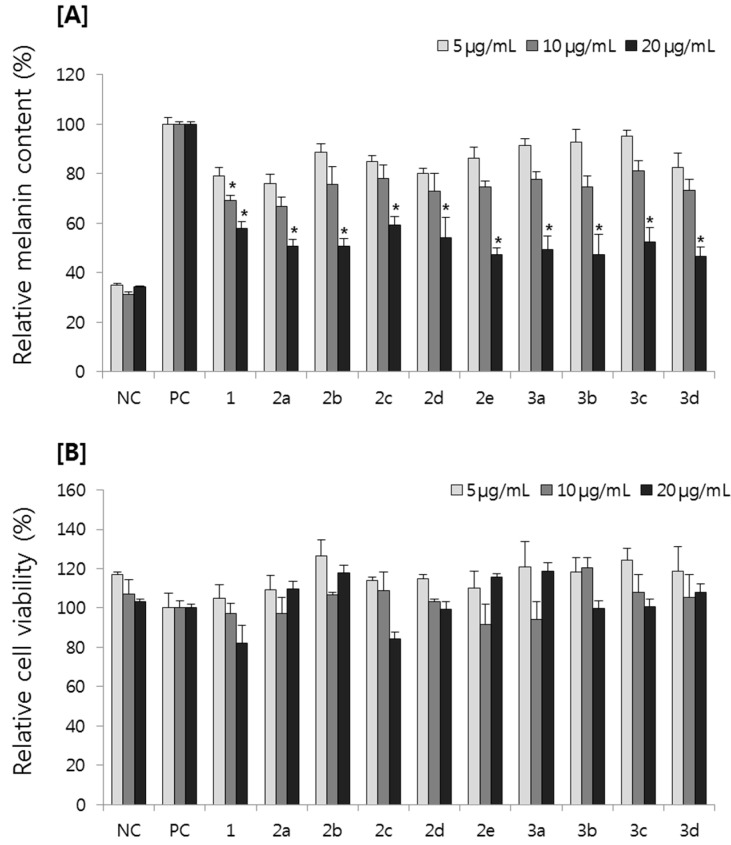
Effects of resveratrol derivatives on (**A**) melanin content and (**B**) cell viability in B16F10 melanoma cells. NC: vehicle treated normal control; PC: α-MSH stimulated positive control. * *p* < 0.05 compared with PC group.

The effect of resveratrol derivatives on melanogenesis and cell viability was first investigated using B16F10 melanoma cells. Stimulation of B16F10 melanoma cells with 100 nM α-MSH for 72 h significantly increased the melanin synthesis. Resveratrol derivatives dose-dependently reduced the melanin content concentration from 5 to 20 µg/mL without any cytotoxicity (Figure 2A,B). Although the inhibition was slightly increased compared to resveratrol in some derivatives, there was no significant difference among resveratrol and synthetic derivatives. In addition, all the synthetic derivatives showed similar inhibition regardless of side chains. 

#### 2.2.2. Effect on Tyrosinase Activity 

Inhibition of melanin synthesis can be achieved either by inhibiting tyrosinase activity or by reducing melanogenic enzyme expression [8,9]. Therefore, the effect of resveratrol derivatives on tyrosinase activity and the expression of melanogenic enzymes were investigated.

**Figure 3:**
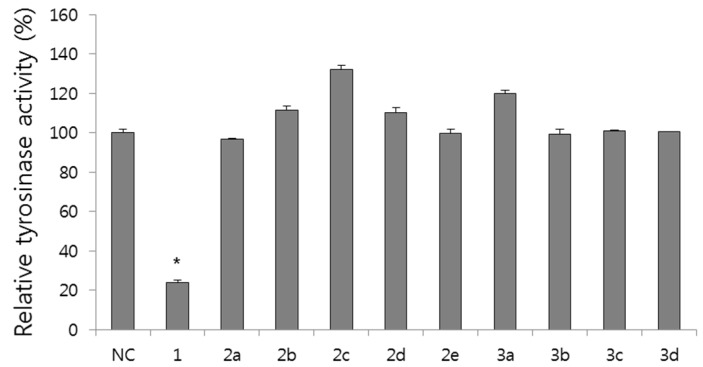
Effects of resveratrol derivatives (100 µg/mL) on tyrosinase activity. NC: vehicle treated normal control. * *p* < 0.05 compared with NC group.

Tyrosinase catalyzes the first rate-limiting step in the melanogenesis and plays a pivotal role in melanin synthesis [6,7]. The effect of resveratrol derivatives on tyrosinase activity was first evaluated *in vitro* using mushroom tyrosinase. Although resveratrol effectively inhibited the tyrosinase activity, both alkyl ether (**2a**–**2e**) and ester derivatives (**3a**–**3d**) showed little inhibition (Figure 3). These results suggest that free hydroxyl groups of resveratrol are important for the inhibition of tyrosinase activity, which is consistent with previous reports [27]. 

#### 2.2.3. Effect on Melanin Synthesis in B16F10 Melanoma Cells

**Figure 4:**
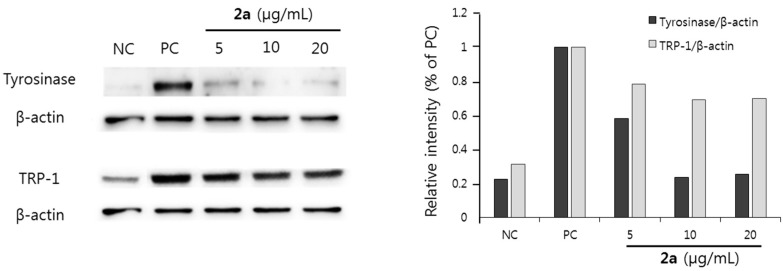
Effect of resveratrol derivative **2a** on the expression of tyrosinase and TRP-1 in B16F10 melanoma cells. NC: vehicle treated normal control; PC: α-MSH stimulated positive control.

Melanin synthesis is also regulated by the expression of melanogenic enzymes. Tyrosinase and TRP-1 are key enzymes involved in the major steps of melanin synthesis [8,9]. Therefore, the effect of the resveratrol derivative **2a** on the expressions of tyrosinase and TRP-1 was determined. The expression of tyrosinase was dramatically reduced by the treatment of compound **2a** (Figure 4). Treatment of **2a** also inhibited the expression of TRP-1 expression. These results suggest that **2a** efficiently inhibited the melanogenic enzyme expression. 

### 2.3. Discussion

Botanical ingredients are good sources of medicine, functional foods and cosmetics. They provide numerous compounds with diverse skeletons and biological activities. However, their applications are often limited due to their small amounts, poor bioavailability, *etc.* Resveratrol is well known for its potential biological activities. As a cosmetic ingredient, it has antioxidant and melanogenesis inhibitory activities. However, it has limitations for cosmetic development, such as chemical instability and low solubility. In addition, the hydroxyl moiety of resveratrol contributes to poor skin absorption. Many attempts have been made to overcome its limitations, and the synthetic derivatives of resveratrol have been suggested as effective in increasing stability and bioavailability [26,28,29,30]. 

In our present study, we synthesized nine resveratrol derivatives, including five ether derivatives (**2a**–**2e**) and four ester derivatives (**3a**–**3d**) and then evaluated melanogenesis inhibitory activity. Our present study showed that all the synthetic ether and ester derivatives of resveratrol inhibited melanin synthesis in melanoma cells. Further study also showed that resveratrol derivative **2a** inhibited melanin synthesis in melanoma cells by inhibiting the expression of melanogenic enzymes, tyrosinase and TRP-1 (Figure 2 and Figure 4). However, it showed little effect on tyrosinase activity (Figure 3). Taken together, we suggest that **2a** reduced melanin synthesis by the inhibition of melanogenic enzyme expressions rather than direct inhibition on tyrosinase activity. 

Skin is a barrier of our body with lipophilic membrane. Therefore, skin absorption of chemical is determined by its physicochemical properties. Lipophilicity is one of the important factors that affect the skin permeation process. Melanocytes and keratinocytes exist in epidermis, the outermost layers of the skin. Thus, lipophilic compounds penetrate deeper in the skin layers [31]. Our synthetic resveratrol derivatives have alkyl or acyl chains instead of free hydroxyl moiety of resveratrol. They exhibit more lipophilic properties than resveratrol, thus these synthetic derivatives are expected to show better bioavailability in skin application. Consistent with our suggestion, triacetyl resveratrol has been reported to have anti-melanogenic activity with better stability [26]. Moreover, resveratrol triacetate reduced hyperpigmented spots in human skin models without any skin irritation [28]. Although the exact mechanism of our synthetic derivatives needs to be confirmed in further animal study, these derivatives might be promising candidates for skin-whitening cosmetics with better practical applications. 

## 3. Experimental Section

### 3.1. General Information

NMR spectra were recorded using a Bruker DRX 500 MHz NMR spectrometer (Bruker, Karlsruhe, Germany). EI-mass spectra were obtained using VG Autospec Ultima mass spectrometers (Waters, Milford, MA, USA). Semipreparative HPLC was performed using a Waters HPLC system equipped with Waters 600 Q-pumps (Waters, Milford, MA, USA), a 996 photodiode array detector Waters, Milford, MA, USA), and Waters Empower software using a Gemini-NX ODS-column (5 μm, 10 mm × 150 mm) (Phenominex, Inc., Torrance, CA, USA). Silica gel (70–230 mesh, Merck, Darmstadt, Germany) was used for open column chromatography (CC). Thin-layer chromatography (TLC) was performed on a precoated silica gel 60 F_254_ (0.25 mm, Merck). All other chemicals and reagents were analytical grade.

### 3.2. Synthesis of Resveratrol Derivatives

*(E)-1,3-Diethoxy-5-(4-ethoxystyryl)benzene* (**2a**). Resveratrol (**1**) (15.0 g, 65.7 mmol) was dissolved in a solution of DMF (150 mL) and NaOH (8.9 g), and stirred for 10 min. Then, 1-bromoethane (25.8 g, 236.6 mmol) was added and the reaction was kept for 24 h at 40 °C. After the removal of DMF by evaporation under vacuum, the reaction mixture was extracted with toluene (45 mL × 2). The combined organic layer were dried over anhydrous MgSO_4_ and evaporated under vacuum. The crude product was purified by silica gel column chromatography with *n*-hexane–EtOAc (95:5) as an eluent to give compound **2a** (15.4 g, 75.0% yield). Compound **2a**: IR ν_max_ cm^−1^: 1587, 1509, 1247; ^1^H-NMR (400 MHz, CDCl_3_) δ 7.45 (2H, d, *J* = 8.4 Hz), 7.05 (1H, d, *J* = 16.4 Hz), 6.91 (2H, *J* = 8.4 Hz), 6.92 (1H, d, *J* = 16.0 Hz), 6.66 (2H, d, *J* = 2.4 Hz), 6.39 (1H, t, *J* = 2.0 Hz), 4.08 (6H, m), 1.45 (9H, m); ^13^C-NMR (100 MHz, CDCl_3_) δ 160.3, 158.8, 139.6, 129.8, 128.6, 127.8, 126.6, 114.7, 104.9, 100.5, 63.5, 14.9, 14.8; ESI-MS (positive mode) *m*/*z*: 313 [M + H]^+^, HRESIMS: *m*/*z* 313.1798 (calcd. for C_20_H_25_O_3_, 313.1798)_._

*(E)-1,3-Dibutoxy-5-(4-butoxystyryl)benzene* (**2b**). Resveratrol (**1**) (15.0 g, 65.7 mmol) was dissolved in a solution of DMF (150 mL) and NaOH (8.9 g), and stirred for 10 min. Then, 1-bromobuthane (30.6 g, 223.5 mmol) was added and the reaction was kept for 24 h at 40 °C. After the removal of DMF by evaporation under vacuum, the reaction mixture was extracted with toluene (45 mL × 2). The combined organic layer were dried over anhydrous MgSO_4_ and evaporated under vacuum. The crude product was purified by silica gel column chromatography with *n*-hexane–EtOAc (95:5) as an eluent to give compound **2b** (21.2 g, 81.5% yield). Compound **2b**: IR ν_max_ cm^−1^: 1589, 1509, 1385, 1294; ^1^H-NMR (400 MHz, CDCl_3_) δ 7.45 (2H, d, *J* = 8.4 Hz), 7.08 (1H, d, *J* = 16.4 Hz), 6.93 (2H, *J* = 8.4 Hz), 6.94 (1H, d, *J* = 16.0 Hz), 6.69 (2H, d, *J* = 2.4 Hz), 6.43 (1H, t, *J* = 2.0 Hz), 4.02 (6H, m), 1.83 (6H, m), 1.57 (6H, m), 1.05 (9H, m); ^13^C-NMR (100 MHz, CDCl_3_) δ 160.5, 159.0, 139.6, 129.8, 128.6, 127.7, 126.6, 114.7, 104.9, 100.5, 67.7, 31.5, 31.4, 19.4, 19.3, 13.9; ESI-MS (positive mode) *m*/*z*: 397 [M + H]^+^, HRESIMS: *m*/*z* 435.2296 (calcd. for C_26_H_36_KO_3_, 435.2296)_._

*(E)-1,3-Bis(pentyloxy)-5-(4-(pentyloxy)styryl)benzene* (**2c**). Resveratrol (**1**) (50.0 g, 219.1 mmol) was dissolved in a solution of DMF (200 mL). After adding NaOH (28.0 g) and KI (1.8 g), the reaction mixture was stirred for 5 min at RT. Then, 1-bromopentane (109.2 g, 223.5 mmol) was added and the reaction was kept for 12 h at 45 °C. After the removal of DMF by evaporation under vacuum, the reaction mixture was extracted with toluene (45 mL × 2). The combined organic layer were dried over anhydrous MgSO_4_ and evaporated under vacuum. The crude product was purified by silica gel column chromatography with *n*-hexane–EtOAc (95:5) as an eluent to give compound **2b** (21.2 g, 81.5% yield). Compound **2c**: IR ν_max_ cm^−1^: 1692, 1385, 1344; ^1^H-NMR (400 MHz, CDCl_3_) δ 7.47 (2H, d, *J* = 8.4 Hz), 7.07 (1H, d, *J* = 16.4 Hz), 6.93 (2H, *J* = 8.4 Hz), 6.94 (1H, d, *J* = 16.0 Hz), 6.68 (2H, d, *J* = 2.4 Hz), 6.42 (1H, brs), 4.00 (6H, m), 1.85 (6H, m), 1.48 (12H, m), 0.99 (9H, m); ^13^C-NMR (100 MHz, CDCl_3_) δ 160.5, 159.0, 139.6, 129.8, 128.6, 127.7, 126.6, 114.7, 104.9, 100.5, 68.0, 29.1, 29.0, 28.3, 28.2, 22.5, 14.1; ESI-MS (positive mode) *m*/*z*: 439 [M + H]^+^, HRESIMS: *m*/*z* 477.2761 (calcd. for C_29_H_42_KO_3_, 477.2765)_._

*(E)-1,3-Bis(hexyloxy)-5-(4-(hexyloxy)styryl)benzene* (**2d**). Resveratrol (**1**) (10.0 g, 43.8 mmol) was dissolved in a solution of DMF (100 mL) and NaOH (5.96 g), and stirred for 10 min. Then, 1-bromohexane (25.8 g, 236.6 mmol) was added and the reaction was kept for 24 h at 40 °C. After the removal of DMF by evaporation under vacuum, the reaction mixture was extracted with toluene (45 mL × 2). The combined organic layer were dried over anhydrous MgSO_4_ and evaporated under vacuum. The crude product was purified by silica gel column chromatography with *n*-hexane–EtOAc (95:5) as an eluent to give compound **2d** (16.3 g, 77.3% yield). Compound **2d**: IR ν_max_ cm^−1^: 1698, 1593, 1457, 1162; ^1^H-NMR (400 MHz, CDCl_3_) δ 7.45 (2H, d, *J* = 8.4 Hz), 7.06 (1H, d, *J* = 16.4 Hz), 6.91 (2H, *J* = 8.4 Hz), 6.92 (1H, d, *J* = 16.0 Hz), 6.67 (2H, d, *J* = 2.4 Hz), 6.42 (1H, t, *J* = 2.0 Hz), 4.00 (6H, m), 1.82 (6H, m), 1.52 (6H, m), 1.39 (12H, m), 0.97 (9H, m); ^13^C-NMR (100 MHz, CDCl_3_) δ 160.5, 158.9, 139.6, 129.8, 128.6, 127.7, 126.6, 114.7, 104.8, 100.5, 68.0, 31.6, 29.4, 29.3, 25.8, 25.7, 22.6, 14.0; ESI-MS (positive mode) *m*/*z*: 481 [M + H]^+^, HRESIMS: *m*/*z* 519.3235 (calcd. for C_32_H_48_KO_3_, 519.3235)_._

*(E)-1,3-Bis(octyloxy)-5-(4-(octyloxy)styryl)benzene* (**2e**). Resveratrol (**1**) (10.0 g, 43.8 mmol) was dissolved in a solution of DMF (100 mL) and NaOH (5.9 g), and stirred for 10 min. Then, 1-bromooctane (25.8 g, 236.59 mmol) was added and the reaction was kept for 24 h at 40 °C. After the removal of DMF by evaporation under vacuum, the reaction mixture was extracted with toluene (45 mL × 2). The combined organic layer were dried over anhydrous MgSO_4_ and evaporated under vacuum. The crude product was purified by silica gel column chromatography with *n*-hexane–EtOAc (95:5) as an eluent to give compound **2e** (21.0 g, 84.7% yield). Compound **2e**: IR ν_max_ cm^−1^: 1694, 1507, 1383, 1298; ^1^H-NMR (400 MHz, CDCl_3_) δ 7.45 (2H, d, *J* = 8.4 Hz), 7.06 (1H, d, *J* = 16.4 Hz), 6.91 (2H, *J* = 8.4 Hz), 6.92 (1H, d, *J* = 16.0 Hz), 6.66 (2H, d, *J* = 2.4 Hz), 6.40 (1H, t, *J* = 2.0 Hz), 4.00 (6H, m), 1.83 (6H, m), 1.50 (6H, m), 1.36 (24H, m), 0.94 (9H, m); ^13^C-NMR (100 MHz, CDCl_3_) δ 160.5, 158.9, 139.6, 129.8, 128.6, 127.7, 126.5, 114.7, 104.8, 100.5, 68.0, 31.8, 29.4, 29.3, 29.2, 26.1, 26.0, 22.6, 14.1; ESI-MS (positive mode) *m*/*z*: 565 [M + H]^+^, HRESIMS: *m*/*z* 603.4176 (calcd. for C_38_H_60_KO_3_, 603.4174)_._

*(E)-5-(4-Acetoxystyryl)-1,3-phenylene diacetate* (**3a**). Resveratrol (**1**) (5.0 g, 21.9 mmol) was dissolved in CH_2_Cl_2_ (40 mL) and kept cool at 10 °C. TEA (7.1 g, 70.1 mmol) and DMAP (0.26 g, 2.19 mmol) were added to the reaction mixture at 10 °C. Acetyl chloride (5.3 g, 67.9 mmol) was added carefully and the reaction was kept for 1 h at 10 °C. After adding distilled water (40 mL) to the reaction mixture, the CH_2_Cl_2_ fraction was extracted. The CH_2_Cl_2_ layers were dried over anhydrous MgSO_4_ and evaporated under vacuum. The crude product was purified by silica gel column chromatography with *n*-hexane–EtOAc (30:1) as an eluent to give compound **3a** (6.9 g, 88.2% yield). Compound **3a**: IR ν_max_ cm^−1^: 1759, 1707, 1532, 1124; ^1^H-NMR (500 MHz, CDCl_3_) δ 7.54 (2H, d, *J* = 8.5 Hz), 7.12 (4H, m), 7.08 (1H, *J* = 16.0 Hz), 6.98 (1H, d, *J* = 16.0 Hz), 6.85 (1H, d, *J* = 2.0 Hz), 2.33 (9H, s); ^13^C-NMR (100 MHz, CDCl_3_) δ 169.3, 169.0, 151.3, 150.4, 139.5, 134.4, 129.6, 127.6, 127.2, 121.9, 116.9, 114.4, 21.1; ESI-MS (positive mode) *m*/*z*: 377 [M + Na]^+^, HRESIMS: *m*/*z* 393.0734 (calcd. for C_20_H_18_KO_6_, 393.0734)_._

*(E)-5-(4-(3-Methylbut-2-enoyloxy)styryl)-1,3-phenylene bis(3-methylbut-2-enoate)* (**3b**). Resveratrol (**1**) (5.0 g, 21.9 mmol) was dissolved in CH_2_Cl_2_ (40 mL) and kept cool at 10 °C. TEA (7.3 g, 72.3 mmol) and DMAP (0.26 g, 2.2 mmol) were added to the reaction mixture at 10 °C. 3,3-Dimethylacroyl chloride (8.3 g, 70.1 mmol) was added carefully and the reaction was kept for 1 h at 10 °C. After adding distilled water (50 mL) to reaction mixture, the CH_2_Cl_2_ fraction was extracted. The CH_2_Cl_2_ layers were dried over anhydrous MgSO_4_ and evaporated under vacuum. The crude product was purified by silica gel column chromatography with *n*-hexane–EtOAc (95:5) as an eluent to give compound **3b** (6.9 g, 92.5% yield). Compound **3b**: IR ν_max_ cm^−1^: 1738, 1645, 1379, 1343; ^1^H-NMR (400 MHz, CDCl_3_) δ 7.50 (2H, dd, *J* = 7.2, 2.0 Hz), 7.11 (4H, m), 7.01 (1H, d, *J* = 16.4 Hz), 6.86 (1H, t, *J* = 2.0 Hz), 2.26 (9H, d, *J* = 1.2 Hz), 2.02 (9H, s); ^13^C-NMR (100 MHz, CDCl_3_) δ 164.7, 164.4, 160.4, 160.1, 151.4, 150.4, 139.3, 134.3, 129.4, 127.5, 127.2, 122.0, 116.8, 115.1, 115.0, 114.8, 27.7, 27.6, 20.6, 20.5; ESI-MS (positive mode) *m*/*z*: 497 [M + Na]^+^, HRESIMS: *m*/*z* 513.1672 (calcd. for C_29_H_30_KO_6_, 513.1673)_._

*(E)-5-(4-(2-Ethylhexanoyloxy)styryl)-1,3-phenylene bis(2-ethylhexanoate)* (**3c**). Resveratrol (**1**) (10.0 g, 43.8 mmol) was dissolved in CH_2_Cl_2_ (50 mL) and kept to cool at 10 °C. TEA (7.3 g, 72.3 mmol) and DMAP (0.54 g, 4.4 mmol) were added to the reaction mixture. After stirring at 10 °C, 2-ethylnexanoyl chloride (22.8 g, 140.2 mmol) was added carefully and the reaction was kept for 1 h at 10 °C. After adding distilled water (50 mL) to reaction mixture, the CH_2_Cl_2_ fraction was extracted. The CH_2_Cl_2_ layers were dried over anhydrous MgSO_4_ and evaporated under vacuum. The crude product was purified by silica gel column chromatography with *n*-hexane–EtOAc (50:1) as an eluent to give compound **3c** (25.2 g, 94.8% yield). Compound **3c**: IR ν_max_ cm^−1^: 1754, 1644, 1376, 1201; ^1^H-NMR (500 MHz, CDCl_3_) δ 7.52 (2H, d, *J* = 8.5 Hz), 7.11 (4H, m), 7.03 (1H, *J* = 16.0 Hz), 6.98 (1H, d, *J* = 16.0 Hz), 6.78 (1H, d, *J* = 2.0 Hz), 2.56 (3H, m), 1.87 (6H, m), 1.76 (8H, m), 1.07 (9H, m), 0.97 (12H, m); ^13^C-NMR (100 MHz, CDCl_3_) δ174.7, 174.4, 151.5, 150.5, 139.4, 134.3, 129.6, 127.6, 127.2, 121.9, 116.8, 114.3, 47.4, 47.3, 31.7, 31.6, 29.6, 25.5, 25.4, 22.6, 13.9, 11.9; ESI-MS (positive mode) *m*/*z* : 629 [M + Na]^+^, HRESIMS: *m*/*z* 645.3550 (calcd. for C_38_H_54_KO_6_, 645.3551)_._

*(E)-5-(4-(Octanoyloxy)styryl)-1,3-phenylene dioctanoate* (**3d**). Resveratrol (**1**) (5.0 g, 21.9 mmol) was dissolved in CH_2_Cl_2_ (40 mL) and kept cool at 10 °C. TEA (7.1 g, 70.1 mmol) and DMAP (0.26 g, 2.2 mmol) were added to the reaction mixture. After stirring at 10 °C, octanoyl chloride (11.0 g, 67.9 mmol) was added carefully and the reaction was kept for 1 h at 10 °C. After adding distilled water (40 mL) to reaction mixture, the CH_2_Cl_2_ fraction was extracted. The CH_2_Cl_2_ layer were dried over anhydrous MgSO_4_ and evaporated under vacuum. The crude product was purified by silica gel column chromatography with *n*-hexane–EtOAc (30:1) as an eluent to give compound **3d** (12.9 g, 96.7% yield). Compound **3d**: IR ν_max_ cm^−1^: 1758, 1644, 1367, 1267; ^1^H-NMR (500 MHz, CDCl_3_) δ 7.51 (2H, d, *J* = 8.5 Hz), 7.11 (4H, m), 7.08 (1H, *J* = 16.0 Hz), 6.99 (1H, d, *J* = 16.0 Hz), 6.82 (1H, d, *J* = 2.0 Hz), 2.58 (6H, m), 1.79 (6H, m), 1.34-1.42 (24H, m), 0.94 (12H, m); ^13^C-NMR (100 MHz, CDCl_3_) δ 172.2, 171.8, 151.4, 150.5, 139.4, 134.3, 129.6, 127.6, 127.2, 121.9, 116.8, 114.4, 34.4, 34.4, 31.6, 29.0, 28.9, 24.9, 24.8, 22.6, 14.0; ESI-MS (positive mode) *m*/*z*: 629 [M + Na]^+^, HRESIMS: *m*/*z* 645.3550 (calcd. for C_38_H_54_KO_6_, 645.3551)_._

### 3.3. Evaluation of Anti-Melanogenesis Activity

#### 3.3.1. Assessment of Tyrosinase Activity 

Tyrosinase inhibitory assays were performed using enzyme solution, which was prepared by the reconstitution of mushroom tyrosinase (Sigma, St. Louis, MO, USA) in 0.1 U/mL phosphate buffer (pH 6.5). A test sample was mixed with 50 μL enzyme buffer, and incubated for 5 min at 37 °C. Then, 50 μL of tyrosine solution, which was diluted with phosphate buffer to 1 mM, was added and the enzyme reaction was allowed to proceed for 20 min at 37 °C. After incubation, the amount of dopachrome formed in the reaction mixture was determined by measuring the absorbance at 490 nm in an ELISA reader (Bio-Tek Synergy HT, Winooski, VT, USA). 

#### 3.3.2. Cell Culture

B16F10 mouse melanoma cells were obtained from the American Type Culture Collection (Manassas, VA, USA). Cells were cultured in Dulbecco’s modified Eagle’s medium (DMEM) supplemented with 10% fetal bovine serum (FBS), 100 IU/mL penicillin and 100 μg/mL streptomycin. Cells were maintained at 37 °C in a humidified atmosphere of 95% air-5% CO_2_.

#### 3.3.3. Measurement of Cellular Melanin Contents

B16F10 cells were stimulated with α-MSH and then treated with resveratrol derivatives at the concentration of 5, 10 and 20 μg/mL for 72 h. After washing with PBS, the cells were harvested and solubilized the melanin by vortexing in 1N NaOH-10% DMSO at 80 °C. The melanin contents were measured by absorbance value at 490 nm with synthetic melanin as a standard.

#### 3.3.4. Cell Viability

B16F10 melanoma were treated with resveratrol derivatives at the concentration of 5, 10 and 20 µg/mL for 72 h. Cell viability was assessed by the 3-(4,5-dimethylthiazol-2-yl)-2,5-diphenyltetrazolium bromide (MTT) assay in an ELISA plate reader. 

#### 3.3.5. Western Blot Analysis 

Stimulated B16F10 cells were treated with resveratrol derivative **2a** for 72 h. Cells were lysed with an SDS lysis buffer containing a protease inhibitor cocktail. The lysates were centrifuged and the supernatants were collected. Proteins were separated in an SDS–polyacrylamide gel and transferred to a PVDF membrane. After blocking in TBST with 5% non-fat dry milk, the membrane was washed and incubated with the primary antibodies including tyrosinase, TRP-1, or β-actin (Santa Cruz Biotechnology Inc., Dallas, TX, USA) at 4 °C overnight. After washing, the membrane was incubated with horseradish peroxidase-conjugated IgG secondary antibody (Cell Signaling Technology Inc., Danvers, MA, USA). The membrane was detected by chemiluminescent reaction, and then exposure to X-ray Kodak film. The proteins of tyrosinase and TRP-1 were normalized by β-actin and the intensities of bands were quantified using a Scion-Image for Windows program (Informer Technologies, Inc., Shingle Springs, CA, USA).

#### 3.3.6. Statistical Analysis 

Evaluation of statistical significance was determined by one-way ANOVA test with a value of *p* < 0.05 considered statistically significant.

## 4. Conclusions 

Nine resveratrol derivatives including five ether derivatives (**2a**–**2e**) and four ester derivatives (**3a**–**3d**) were newly synthesized and evaluated for melanogenesis inhibitory activity. All the synthetic ether and ester derivatives of resveratrol inhibited melanin synthesis in melanoma cells. Further Western blot analysis showed that resveratrol derivative **2a** inhibited melanin synthesis by the inhibition of melanogenic enzyme expressions rather than direct tyrosinase activity. Our synthetic resveratrol derivatives have more lipophilic properties than resveratrol by the addition of alkyl or acyl chains to free hydroxyl moiety of resveratrol, thus are expected to show better bioavailability in skin application. Taken together, we suggest that our synthetic resveratrol derivatives might be promising candidates for better practical application to skin-whitening cosmetics.
